# Attendance in physical education classes, sedentary behavior, and different forms of physical activity among schoolchildren: a cross-sectional study

**DOI:** 10.1186/s12889-022-13864-9

**Published:** 2022-08-01

**Authors:** Gilmar Mercês de Jesus, Raphael Henrique de Oliveira Araujo, Lizziane Andrade Dias, Anna Karolina Cerqueira Barros, Lara Daniele Matos dos Santos Araujo, Maria Alice Altenburg de Assis

**Affiliations:** 1 State University of Feira de Santana, Feira de Santana, BA Brazil; 2 State University of Londrina, Postgraduate Program in Health Sciences, Londrina, PR Brazil; 3 Federal University of Santa Catarina, Florianópolis, SC Brazil

**Keywords:** Physical education and training, Motor activity, Sedentary behavior, Child

## Abstract

**Background:**

Attendance in physical education classes (PE) helps young people to achieve the physical activity recommendations and to reduce their exposure to sedentary behavior. However, the association between PE attendance and the daily frequency of specific forms of physical activity is less known. The current study analyzed the association between weekly attendance in PE and daily frequencies of different forms of physical activity (active play, non-active play, structured physical activity), and overall daily frequencies of physical activity (PA) and sedentary behaviors (SB) among schoolchildren.

**Methods:**

Cross-sectional study with schoolchildren from second to fifth grade of 11 public schools (*n* = 2,477; 9.1 ± 1.38-y-old; 53.2% girls; 17.5 ± 3.5 kg/m^2^) in Feira de Santana (Northeast Brazil). PA, SB, and attendance in PE were self-reported using a previously validated on-line questionnaire based on the previous day's recall (Web-CAAFE). Multiple Binomial Negative regression modeling was carried out to analyze the association (Prevalence Rate: PR) between weekly attendance in PE (0/week, 1/week, ≥ 2/week) and frequencies of active play, non-active play, and structured physical activity, with adjustments by age, school shift, and BMI z-scores. Regression models analyzing overall PA also included adjustments by household chores.

**Results:**

Attendance in PE ≥ 2/week was associated with higher frequencies of active play (girls: PR = 1.40, 95%CI = 1.11–1.78; boys: PR = 1.49, 95%CI = 1.15–1.94) and structured physical activity (girls: PR = 2.11, 95%CI = 1.31–3.40; boys: PR = 4.33, 95%CI = 1.63–11.52). Higher attendance in PE (≥ 2/week) was associated with high overall PA (girls: PR = 1.31, 95%CI = 1.06–1.62; boys: PR = 1.42, 95%CI = 1.14–1.77) and low SB (girls: PR = 0.80, 95%CI = 0.71–0.90; boys: PR = 0.81, 95%CI = 0.68–0.97). Attendance in PE 1/week was also associated with a lower frequency of daily SB among girls (PR = 0.73, 95%IC = 0.64–0.84)

**Conclusion:**

Higher weekly attendance in PE was associated with higher frequencies of active play, structured physical activity, higher overall PA, and lower SB among both girls and boys.

## Background

Physical education is an academic discipline that covers biomechanical, psychological, cultural, and social aspects of human movement, and needs to be integrated into the school curriculum as a fundamental aspect for the development of children and adolescents [1]. In Brazil, physical education classes are required to be offered in basic education, an educational stage that covers early childhood education (children from 0 to 5 years old), elementary school (from 1st to 9th grade), and high school [2].

Participating in physical education classes (PE) allows young people to gain knowledge and develop skills to engage in sports, games, dances, and fight modalities [3]. Furthermore, PE contributes to the achievement of physical activity recommendations [4–9] while helping to reduce the exposure to sedentary behavior (SB)[4–6]. In general, these effects can be acquired through participation in at least one physical education class per week [8, 10, 11], but the gains can be even greater with more weekly classes [7, 8].

The scope of physical activity recommendations and reductions in exposure to daily SB among children and adolescents are relevant issues for education and health decision makers [10]. The regular participation of children and adolescents in physical activity improves cardiorespiratory fitness [11, 12], muscle, and bone health, and may play a key role in preventing and controlling obesity [10, 13]. In addition, physical activity has positive effects on executive functions, attention, and academic performance [14]. Excessive SB during childhood and adolescence are of concern because long periods of watching TV and using computers and video games are associated with decreased cardiorespiratory fitness, unfavorable cardiovascular outcomes [15], and an inadequate diet [16].

Despite this, globally, 81% of adolescents are insufficiently active [17]. In Brazil, this prevalence reaches 83.6%, ranging from 78% to 89.4% among boys and girls, respectively [17]. In addition, 58.8% of Brazilian adolescents exceeded the SB recommendations, when evaluated through time watching TV [18]. Given this scenario, the World Health Organization recommends that all countries should allocate resources for the implementation of policies with an integrated and systemic approach, with the objective of promoting physical activity in a diversified manner, including improving access to public outdoor spaces, from infrastructure and road safety to promoting active commuting, offering active sports and games programs, promoting physical activity at school, and increasing physical education [19].

Physical education in school is an effective and inclusive way of providing the skills for lifelong participation in physical activity and sport [20]_._ Indeed, many studies present the beneficial effects of a higher weekly frequency of participation in physical education classes on SB and physical activity [4–9, 12, 21–26]. However, as far as we know, previous studies did not analyze the association between attendance in PE and specific forms of physical activity (e.g., different sports and active play) or sedentary behavior (e.g., use of screens). Information based on specific forms of physical and sedentary activities is more easily understood and able to be used by physical education teachers. Furthermore, this information could provide a comprehensive view of the effectiveness of physical education classes to develop the skills and knowledge necessary for children to practice different forms of physical activity, such as sports, active games, and fight sports. Through long-term monitoring of forms of physical activity, we can detect changes in young people's preferences and the contexts and places that are more conducive to different forms of physical activity and SB.

Based on these aspects, the primary objective of the current study was to analyze the association between weekly attendance in PE and different forms of physical activity. We also include an analysis of this association by daily frequencies of physical activity (DPA) and SB.

## Methods

### Participants

**Fig. 1:**
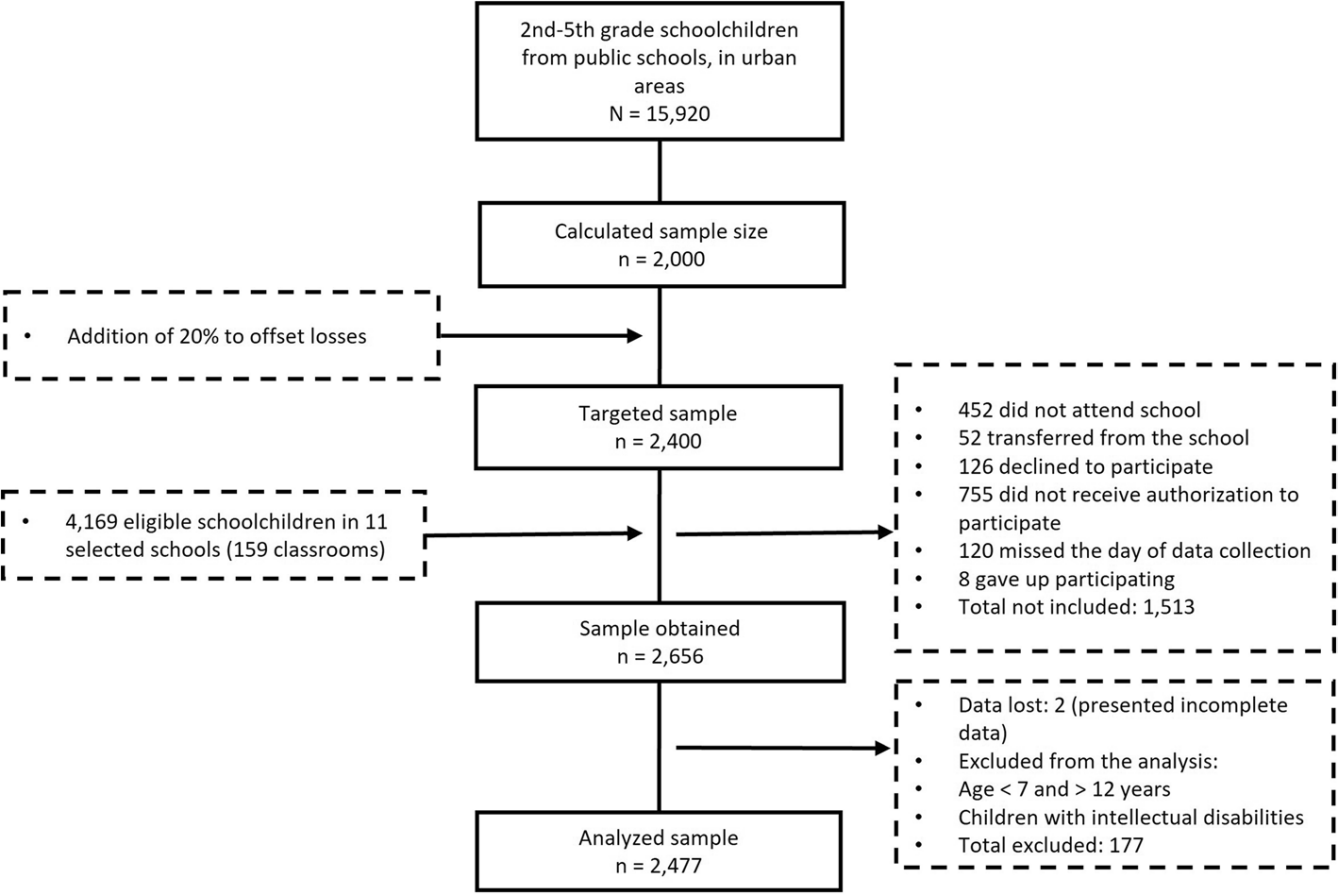
Study flowchart

Schoolchildren (7–12 years-old) from 2nd to 5th-grade in part-time public schools in Feira de Santana (Bahia) participated in this cross-sectional study. Feira de Santana is in the Northeast region of Brazil (inhabitants: 624,107; Human Development Index: 0.712). Data collection covered weekdays (Tuesday to Friday), from March to October of the year 2019 and included a probability sample of students from 2nd to 5th-grade, from public schools in the urban area, with broadband Internet. The sample size was defined based on the following parameters: a population of 15,920 students enrolled in the education system, according to data from the Municipal Department of Education; expected prevalence of outcomes of 50%; confidence limit of three percentage points; design effect (*deff*) of 2.0; and 95% confidence interval (95%CI). Based on these parameters, the sample size was calculated at 2,000 students. A further 20% was added to make up for presumed losses, resulting in a sample of 2,400 students (Fig. 1).

The cluster sampling process was carried out in three stages: I) all schools in the municipal network were stratified according to the 11 geographic and administrative centers of the Department of Education (clusters); II) one school from each center was randomly drawn; III) all classrooms from 2^nd^ to 5^th^ grade within each school were selected (159 classrooms), and all subjects within the selected classrooms were invited to participate in the study. All methods were carried out in accordance with relevant guidelines and regulations of ethical standards set out in Resolution No. 466/2012 of Brazil’s National Research Ethics Council. Informed consent was obtained from all participants involved in the study and their parents/guardians provided authorization in writing. The study protocol was approved by the Research Ethics Council of the State University of Feira de Santana (Approval No. 02307918.5.0000.0053, Opinion No.: 3.116.495). The Municipal Department of Education provided information regarding the sex, age, and school shift of participants.

### Measurement of sedentary behaviors and physical activities

The participants self-reported the SB and physical activity on the Food Intake and Physical Activity of Schoolchildren (Web-CAAFE) questionnaire. The Web-CAAFE is a previously validated self-report questionnaire [27], completed on the internet and based on the previous-day recall. The instrument collects information on weight status, food consumption, physical activity, and SB and includes screens to evaluate physical education classes and to investigate modes of commuting to school.

Participants choose up to 32 items, out of a total of 50 stored in the system, which they had performed the day before across three periods (morning, afternoon, evening). The list contains five SB icons (one for academic tasks, e.g. reading, writing, drawing, painting; four electronic devices, e.g. TV, video game, computer, and cell phone), and 27 physical activity icons classified into: Active play (Play with a ball, Play catch, Soccer, Dance, Marbles, Jump rope, Gymnastics, Elastics, Play in the park, Play in the water/Swim, Ride a bicycle, Rollerblade/Skateboard/Ride a scooter, Fly a kite, Dodgeball, Hide and seek, Play with a dog, Hopscotch), Non-active play (Board games, Playing with dolls/action figures, Playing with toy cars, Spinning top/Bayblade, Listen to music, Play musical instrument), Structured physical activity (Ballet, Fight Sports), and Household chores (Wash the dishes, Sweep). Information on the weekly frequency of participation in physical education classes is assessed through the question *“How many times a week do you take part in physical education classes?*” (none, 1, 2 3, 4, every day of the week). The closed list of leisure activities, sports, home chores, and sedentary activities was compiled based on results from focal groups, previous instruments for this age range, and the 7-day recall completed by 180 schoolchildren [28].

Participants completed the Web-CAAFE at the school, after receiving verbal explanations about how the software works and how to complete the questionnaire. Students were instructed not to interact during the task and the research team helped when requested, without inducing responses.

### Anthropometric measurements

The study included weight and height measurements to calculate the Body Mass Index (BMI), measured by trained researchers, following recommended standardization [29]. Weight was measured using an AVAnutri® digital scale with graduation every 100 g and a maximum capacity of 200 kg. Height was measured using a portable stadiometer, detachable, with a square platform, Seca® brand, with a 205 cm maximum height and graduation every 1 mm. The students were barefoot, wearing school uniform, and with no headwear during measurements. Age-and sex-specific BMI z-scores were calculated according to the International Obesity Task Force (IOTF) [30]. The weight status was categorized into non-overweight (underweight and normal weight), overweight, and obesity according to IOTF reference values [30].

### Classification of economic level

Socioeconomic status was investigated based on the analysis of possession of items, education level of the head of the household, and access to public services, according to the Brazilian Economic Classification Criteria [31]. The socioeconomic status was classified into classes, related to the average household income in Reais (R$): A (R$25,554.33), B-C (R$1,748.59 to R$11,279.14), and D-E (R$719.81). Based on the average dollar exchange rate between March and October 2019, income ranges in these classes were: A (US$ 6,485.87), B-C (US$ 443.80 to 2,862.72), and D-E (US$ 182.69).

### Data processing and analysis

The weekly attendance in PE was the main exposure analyzed (0/week; 1/week; ≥ 2/week). Daily frequencies of active play, non-active play, and structured physical activity were the main outcomes (count outcomes). These frequencies were obtained by summing all reports in the morning, afternoon, and night. For example, if a participant reported riding a bike in the morning period, playing with a ball in the afternoon, and playing with a dog in the evening, then their sum was 3 counts of active play. SB frequency was obtained by summing the daily reports of academic tasks and screen use. DPA frequency was obtained by summing the daily reports of all physical activities.

Students with intellectual disabilities and ages outside the age group of seven to 12 years participated in the study but were excluded from the statistical analyses. Descriptive statistics are used to present the study variables. Variables without normal distribution after verification of the histograms and the Shapiro–Wilk test are described by median and interquartile range values. Differences in non-normally distributed continuous variables were evaluated using the non-parametric Mann–Whitney test (*U*). Categorical variables are described as absolute and relative values and compared using Pearson's chi-square test (*Χ*^2^).

The associations between weekly attendance in PE and frequencies of active play, non-active play, and structured physical activity were analyzed using the values of prevalence ratios (PR) and respective 95%CI estimated via multiple Negative Binomial Regression, with adjustment for age (7–9 years; ≥ 10 years), school shift (morning; afternoon), and BMI *z*-scores, adopting a robust variance estimation method. Negative Binomial models analyzing the association between weekly attendance in PE and DPA and SB were also adjusted by the daily frequency of household chores. The group of household chores was not included in the present analysis as an outcome because there is no evidence of an association with attendance in PE.

The Negative Binomial distribution is suitable for fitting count data susceptible to overdispersion. In addition, it showed higher linearity in the comparison between observed and predicted values of the outcome. The zero-inflation between the factors was assumed to be constant. Although the negative binomial regression models provide a measure of association such as Incidence-Rate Ratios (IRR), we adopted the prevalence ratio (PR) as the most appropriate way to present our results, considering the cross-sectional design of the study. Statistical significance was assessed using *p* value < 0.05. Effect modification was tested using interaction terms between weekly attendance in PE and sex, age, school shift, and BMI *z*-scores. Interactions that showed statistical significance at the critical value of *p* < 0.05 were described.

## Results

The study included 2,654 children who agreed to participate and were authorized by their parents/guardians. Of these, 177 participants who presented intellectual disabilities and with ages outside the range of seven to 12 years old were excluded from the analysis. Based on these criteria, the analytical sample consisted of 2,477 children {Age(mean ± SD) 9.1 ± 1.38 years old; BMI(mean ± SD) 17.5 ± 3.5 kg/m^2^}.

The characteristics of the sample are presented in Table 1. The proportions of girls and boys did not differ in terms of school shift, age, and weight status. Although the majority of the participants reported attending PE 1/week, approximately 9% of the sample reported not attending any weekly PE and this was more pronounced among girls. Boys presented more reports of PE ≥ 2/week than girls. The socioeconomic status assessment questionnaire had a low response rate, with missing data ranging from 35% to 40.3% in all items. Thus, it was only possible to classify the economic level of 972 participants (39.2%), who were concentrated between classes B-C and D-E, with no statistical differences between boys and girls. Considering the large amount of missing data, the economic level was not included in the statistical modeling, via multiple Binomial Negative regression. No other missing data were observed.

**Table 1:** Sample characteristics

Variables	Girls N (%)	Boys N (%)	*p* value
School shift			
Morning	680 (51.6)	593 (51.1)	0.799^†^
Afternoon	637 (48.4)	567 (48.9)	
Age			
7–9 years	800 (60.7)	705 (60.8)	0.987^†^
10–12 years	517 (39.3)	455 (39.2)	
Socioeconomic stratum^a,b^			
A (R$25,554.33)	8 (1.5)	2 (0.4)	0.100^†^
B-C (R$1.748,59 a R$11,279.14)	349 (66.5)	291 (65.1)	0.652^†^
D-E (R$719.81)	168 (32.0)	154 (34.5)	0.418^†^
Weight status			
Low weight/normal weight	1,033 (78.4)	951 (82.0)	0.027^†^
Overweight	192 (12.2)	141 (14.6)	0.078^†^
Obesity	92 (7.0)	68 (5.9)	0.256^†^
Attendance in Physical education classes (PE/week)			
0/week	130 (11.1)	87 (8.3)	0.037^†^
1/week	689 (58.9)	566 (54.1)	0.022^†^
≥ 2/week	351 (30.0)	394 (37.6)	< 0.001^†^
Active play^c,d^	1 (0—16)	2 (0—16)	< 0.001^‡^
Non-active play^c,d^	0 (0—6)	0 (0—5)	0.001^‡^
Structured physical activity^c,d^	0 (0—4)	0 (0—3)	0.010^‡^
Daily physical activity (DPA)^c,d^	3 (0—25)	3 (0—17)	0.804^‡^
Daily Sedentary behavior (SB)^c,d^	2 (0—11)	2 (0—10)	0.045^‡^

^a^Information available for 972 participants (525 girls and 447 boys). ^b^Socioeconomic stratum (average household income in R$). ^c^median (minimum value – maximum value. ^d^Sum of all daily reports on previous day (morning, afternoon, evening). ^†^Pearson's Chi-square test $$\left({\upchi }^{2}\right)$$. ^‡^Mann–Whitney test (U)

Sex, age, school shift, and BMI *z*-scores did not modify the effect of weekly attendance in PE on daily frequencies of DPA or SB. Even so, we chose to carry out the analyses stratified by sex, considering the differences in movement behavior between boys and girls, especially regarding the different forms of physical activity and SB.

Table 2 presents the associations of weekly attendance in PE with SB, DPA, active play, non-active play, and structured physical activity. Girls attending 1 PE/week and ≥ 2 PE/week presented, on average, 27% and 20% less SB compared to their peers who reported attending no PE classes. Among boys, only those attending ≥ 2 PE/week exhibited 19% less SB than those attending no PE classes. On the other hand, PE was positively associated with DPA, which was 31% and 42% higher, respectively, among girls and boys attending ≥ 2 PE/week, compared to their peers who did not attend PE. Compared to schoolchildren who reported not attending PE, those reporting attending ≥ 2 PE/week presented a significantly higher probability of reporting active play (girls 40%; boys 49%). Girls attending ≥ 2 PE/week presented, on average, a probability twice as high for structured physical activity than their peers with no PE. Compared to boys who reported no PE/week, those reporting attending ≥ 2 and 1 PE/week presented, respectively, four and three times higher probability for participation in structured physical activity. No association was observed between attendance in PE and non-active play.

**Table 2:** Association between attendance in physical education classes, sedentary behaviors, and physical activities among children and adolescents. Results presented through prevalence ratios (PR) and respective 95% Confidence Intervals (95%CI)

Attendance in Physical education classes (PE/week)	Daily Sedentary Behavior^†‡^	Daily Physical Activity^†‡d^	Active Play^†‡^	Non-active Play^†‡^	Structured Physical Activities^†‡^
	**Girls (*n* = 1,317)**				
≥ 2/week	0.80 (0.71–0.90)^a^	1.31 (1.06–1.62)^c^	1.40 (1.11–1.78)^b^	0.79 (0.57–1.10)	2.11 (1.31–3.40)^b^
1/week	0.73 (0.64–0.84)^a^	0.95 (0.77–1.17)	0.97 (0.77–1.22)	0.63 (0.85–1.23)	1.10 (0.68–1.76)
0/week	1.00	1.00	1.00	1.00	1.00
	**Boys (*n* = 1,160)**				
≥ 2/week	0.81 (0.68–0.97)^c^	1.42 (1.14–1.77)^b^	1.49 (1.15–1.94)^b^	0.95 (0.68–1.34)	4.33 (1.63–11.52)^b^
1/week	0.94 (0.80–1.10)	1.18 (0.95–1.46)	1.20 (0.92–1.55)	0.83 (0.59–1.16)	3.17 (1.19–8.47)^c^
0/week	1.00	1.00	1.00	1.00	1.00

^a^*p* < 0.001. ^b^*p* < 0.01. ^c^*p* < 0.05. ^†^Sum of all daily reports on previous day (morning, afternoon, evening). ^‡^PR and 95%CI estimated via multiple Binomial Negative Regression modeling with adjustment by age, school shift, and sex-and-age specific BMI z-scores according to cut-off points of the International Obesity Task Force (IOTF). ^d^Modeling adjusted by daily frequency of household chores

## Discussion

The current study investigated the association between weekly attendance in PE and the frequencies of different forms of physical activity among schoolchildren from public schools. Our results demonstrated that a higher weekly attendance in PE (≥ 2/week) was associated with active play and structured physical activity among girls and boys. The non-active play was negatively associated with age (10–12 years) among boys. Furthermore, boys attending PE ≥ 1/week also exhibited a higher frequency of structured physical activity. Overall, both girls and boys with a higher weekly attendance in PE (≥ 2 /week) presented a higher frequency of DPA and, at the same time, a lower frequency of SB. Attending PE ≥ 1/week was associated with a lower frequency of SB only among girls.

Evidence from previous studies showed that girls benefited more from having two or more weekly PE compared to boys, although this result was more consistent for children [6] than for adolescents [7–9].

Studies about forms of physical activity and SB during childhood are important because these behaviors can persist into adolescence and adulthood [32], when they constitute risk factors for chronic non-communicable diseases [33]. On the other hand, participation in PE during youth is associated with positive outcomes in adult life, including benefits to metabolic health, perceived health, physical activity, and cardiorespiratory fitness [34].

However, comparisons between our results and those from the available scientific literature should be performed with caution, since the outcomes analyzed in our study were the self-reported frequencies of specific forms of physical activity and SB, while other studies analyzed objective measurements of time spent in moderate to vigorous physical activity (MVPA) [4–6, 22, 26] or self-reported measures in order to assess compliance with the recommendation of 60 min daily of MVPA [7–9, 12, 21, 23–26]. In our study, we used a health surveillance tool, the purpose of which is to qualitatively assess the forms of physical and sedentary activities and provide a description of the total frequency of activities reported on the previous day, as a proxy for the physical activity level of the participants[27]. Taking these aspects into account, we can state, at least partially, that our results corroborate previous studies that showed the association between participation in PE with higher levels of physical activity and lower levels of SB among children and adolescents [4–9].

PE contributes to an important percentage of the physical activity of school-age children and adolescents and, in specific contexts, can represent the main or only opportunity for students to participate in physical activity [25]. In addition, PE allows young people to become familiar with different forms of physical activity and sports and develop skills to practice them and this can encourage them to be physically active in environments outside of school [6]. On the other hand, previous studies have shown that adolescents who are more active in out-of-school environments are also more interested in participating in PE, as they feel more confident and self-effective to engage in physical activities and sports [12, 25]. These findings suggest the existence of a bidirectional association between PE and physical activity, an aspect that may have influenced the results of the present study, since the outcomes included all physical activities reported in the morning, afternoon, and night, covering activities performed in and out of school. Indeed, our results showed that girls and boys with a higher weekly attendance in PE exhibited a higher frequency of structured physical activity (e. g., soccer, fight sports, and ballet). It is important to highlight that our outcome included all physical activities performed throughout the day and this may have overlapped those performed in the PE class itself. However, our objective was not to analyze the association between activities performed in PE and those performed during leisure time and this analysis could be a way to investigate the bidirectional association hypothesis in further research.

Our results also showed that attendance in PE did not influence the participation in non-active play. The Web-CAAFE is a tool developed and validated in Brazil and contains a list of icons that represent habitual children’s movement behaviors over the course of a day. Since the questionnaire is not limited to evaluating only the moment of the physical education class, the non-active games included in the icons list cannot be traditionally taught in PE in the study setting (e.g., board games, playing with dolls/action figures, playing with toy cars, spinning top/Beyblade). Thus, it was expected that they would not present an association with participation in physical education classes.

The present study was carried out in a region of the country with a lower HDI (113th place in the national ranking of cities) and that only included public school students. These schools cater to families with a lower income, with more restricted access to private places for exercise and sports, and who live on the outskirts of cities where there is a greater perception of insecurity. These factors generate barriers to the practice of physical and sports activities in leisure time and, thus, PE can represent an important part of the opportunities for young people to be physically active and accumulate less SB.

Based on the aforementioned, our study highlights the importance of PE as an appropriate environment to promote physical activity. In addition, our findings can be used to support local policies to reinforce PE in schools, ensuring that classes are offered (approximately 10% of participants reported having no weekly classes) and the expansion of the weekly number of classes (approximately 57% of participants reported having classes only once a week), so that the benefits of reducing daily SB and increasing PA can be achieved by a greater number of young people. It is important to highlight that although the offer of physical education classes is provided in Brazilian educational laws, this is not always respected by the municipalities of the federations that are managers of the public elementary education network. Indeed, we interviewed the principals of the participating schools and two of them reported that their schools did not offer physical education classes. Moreover, in another school, participation in physical education classes could be replaced by another activity outside the school (e.g., ballet, sports, capoeira, judo, karate, or gymnastics classes). These aspects help to explain why there are students who did not attend physical education classes in our sample.

As far as we have been able to investigate, our study is the first in Brazil to analyze the association between participation in PE and different forms of physical activity among children. In addition, our sample is representative of students from public elementary schools in the city and does not include students from private schools.

Some limitations are worth mentioning. First, as this is a cross-sectional study, it is not possible to make a causal inference. Socioeconomic data were not available for all participants, so it was not possible to analyze the effect of economic class on the tested associations without losing the power of the sample. However, the distribution of researched subjects according to economic class was similar to that presented for the Northeast region of Brazil [31]. It is important to highlight that the loss of socioeconomic information has also been observed in previous works with Brazilian students who answered questionnaires in the classroom and under the supervision of the research team [12, 25], although no effect of the economic level on the researched outcomes was noted. Unlike those studies, we chose to send the forms to parents or guardians, since our sample consisted of a significant number of children in the literacy phase, which may have influenced the losses recorded. Future studies should make efforts to avoid loss of this information, by adapting the data collection procedure for children and adolescents. For instance, if data are available from the Brazilian Institute of Geography and Statistics (IBGE), the average census sector income of the school location area could be used as a proxy for the family income, as the family residential address determines the school a child is assigned to [35]. In addition, the use of a question on food security status could be promising as a proxy of family income, such as that used by the Global School-based Student Health Survey (GSHS) [36].

Furthermore, in our study, physical activity and SB were assessed through self-report, which is subject to memory and social desirability bias. However, the Web-CAAFE was validated with schoolchildren from the city where this research was carried-out. The instrument relies on recall of the previous day, which is less susceptible to recall bias compared to instruments that rely on a longer recall period (e.g. last week, last month, last year). The cognitive task required for estimating frequency and averaging may not be compatible with the perceptual and conceptual capacities of children who have not reached the stage of abstract reasoning, at approximately 10–11 years of age[37]. Although objectively measured physical activity data are recommended in studies including children, this requires considerable financial resources, so is not always feasible in many countries. Thus, it is advisable to use the best available evidence to support and guide actions to promote physical activity, even if based on the reports of the subjects themselves[38]. Future studies could consider the use of wrist-worn wearables to provide additional objective information on physical activity, which are less expensive devices and which can provide measurements with acceptable validity and reliability [39, 40]. However, more evidence is needed on the application of these devices for the assessment of children.

## Conclusions

Higher weekly attendance in PE was associated with higher frequencies of active play, structured physical activity, and DPA, and lower SB, overall, among both girls and boys. This finding is relevant and supports the arguments to reinforce the importance of PE, as an essential aspect for the healthy development of children. It is also important to highlight the role that schools can play in reducing inequities in physical activity, since PE offered in public schools may be one of the few spaces for children to have access to sports activities.

## Data Availability

The datasets generated and/or analyzed during the current study are not publicly available due to lack of authorization from the Research Ethics Council but are available from the corresponding author upon reasonable request.

## Abbreviations

95%CI: 95% Confidence Intervals
BMI: Body Mass Index
DPA: Daily physical activity
HDI: Human Development Index
IOTF: International Obesity Task Force
IRR: Incidence-Rate Ratios
MVPA: Moderate to vigorous physical activity
PE: Physical education classes
PR: Prevalence Ratio
SB: Sedentary behavior
Web-CAAFE: Food Intake and Physical Activity of Schoolchildren Questionnaire
